# The concentration data of fluoride and health risk assessment in drinking water in the Ardakan city of Yazd province, Iran

**DOI:** 10.1016/j.dib.2018.02.069

**Published:** 2018-03-06

**Authors:** Majid Mirzabeygi (Rad Fard), Mahmood Yousefi, Hamed Soleimani, Ali Akbar Mohammadi, Amir Hossein Mahvi, Abbas Abbasnia

**Affiliations:** a Department of Environmental Health, School of Public Health, Tehran University of Medical Sciences, Tehran, Iran; b Student's Scientific Research Center, Tehran University of Medical Sciences, Tehran, Iran; c Ava Salamat Entrepreneurs Institute, Tehran, Iran; d Center for Solid Waste Research, Institute for Environmental Research, Tehran University of Medical Sciences, Tehran, Iran; e Department of Environmental Health Engineering, Neyshabur University of Medical Sciences, Neyshabur, Iran

**Keywords:** Fluoride, Groundwater, Health risk, Ardakan, Iran

## Abstract

According to the World Health Organization (WHO) reports, Iran is located in the global fluoride belts, so that is why carrying out extended research on this contaminant anion in water supplies must be considered. Due to the various industries in the Ardakan city, this region is severely suffering from fluoride contamination. This study was designed to investigate the fluoride concentration and its distribution pattern as well as related health risk assessment in groundwater resources of 28 villages of the Ardakan city in Yazd province using SPADNS method according to standard instructions. Our results show that, the average concentration of fluoride was 2.92 mg/l (range: 0.9–6 mg/l), also in half of the villages, the concentration range of this element was over than standard level (1.5 mg/l) given by WHO rules. In addition, risk assessment results showed that HQ value is higher than 1 in 46.4% of samples of groundwater resources in age groups of infants, children, teenagers and adults. Therefore, it is necessary to take measures to reduce fluoride concentration in drinking water in order to control resultant fluorosis. Actions should be implemented to enhance monitoring of fluoride levels to avoid the potential risk of high Fluoride concentration.

## Specifications table

**Table:** 

Subject area	Water quality
More specific subject area	Water fluoride
Type of data	Tables, Figures
How data was acquired	Analysis of all water samples were done according to the Standard Methods for Examination of Water and Wastewater. Anions and cations including magnesium, calcium, and chloride as well as temporary and permanent hardness were measured using titration method. Turbidity meter (model Hach 50161/co 150 model P2100Hach, USA) was used to analysis of electrical conductivity. Also, determining of fluoride, nitrate, and sulfate concentration in comparison with internal standards were done using Hach DR5000 spectrophotometer.
Data format	Raw, Analyzed
Experimental factors	All water samples were stored in polyethylene bottles in a dark place at room temperature until analysis.
Experimental features	Determine the concentration levels of fluoride
Data source location	Ardakan region, Yazd province. Iran
Data accessibility	Data are included in this article

## Value of the data

• Based on health risk assessment, and data analysis we found that HQ amounts exceeded standard levels, and therefore defluoridation of drinking water could be recommended in subjected region.
• In order to minimize of fluoride in point of use (POU) and point of entry (POE), urgent actions needed to be implement to address them is necessary.
• The Iranian standard of fluoride in drinking water is based on the maximum annual temperature of the area because of temperature impact on water evaporation.

## 1 Data

**Fig. 1:**
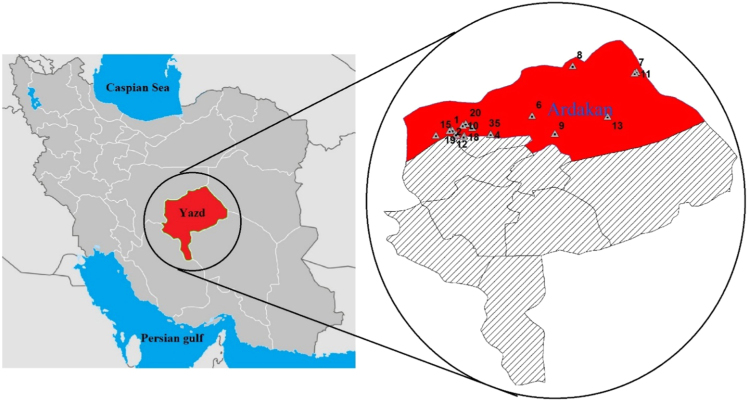
Groundwater water sampling sites in Ardakan.

**Fig. 2:**
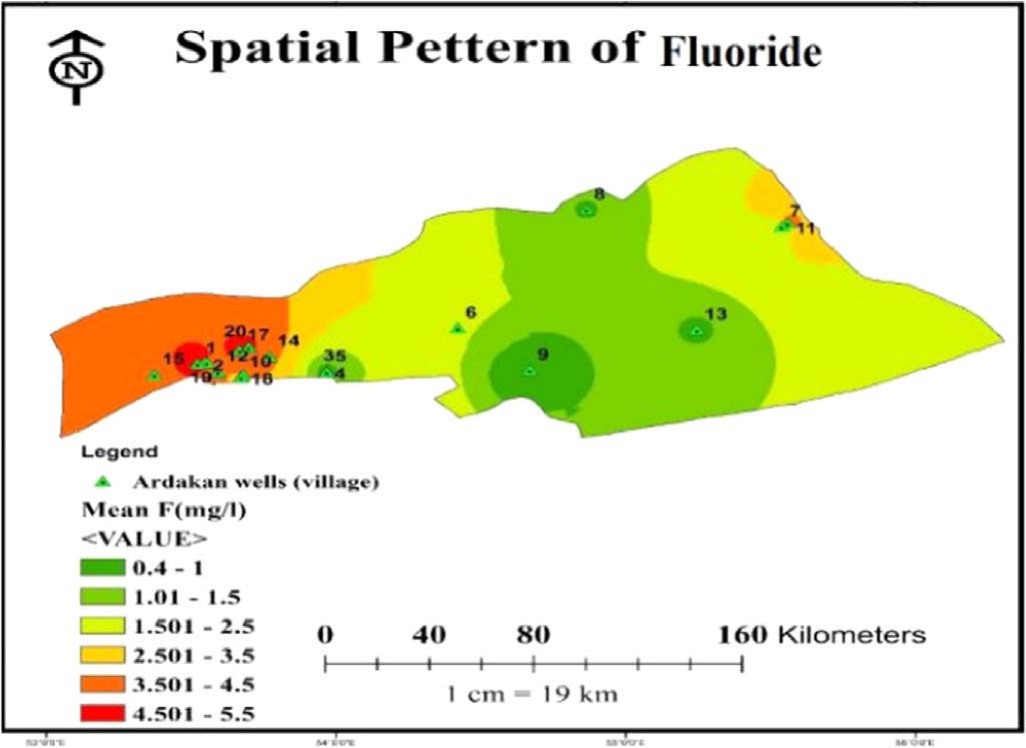
Geological distribution of fluoride in study area.

**Fig. 3:**
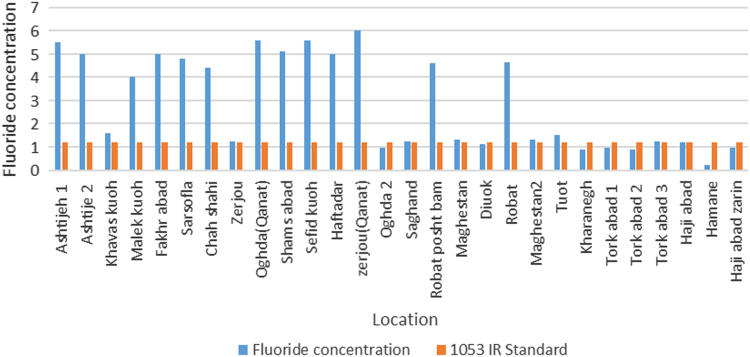
Comparison of fluoride concentration with 1053IR standard.

Concentration of studied physicochemical parameters in the groundwater of the 28 villages and towns of the Ardakan region are summarized in Table 1 and Fig. 1. Also geological distribution of fluoride in the study area is also illustrated in Figs. 2 and 3 and also Fig. 3 comparison of fluoride concentration with 1053IR standard. In addition, the correlation between the all parameters is shown in Table 2.

**Table 1:** Physico-chemical and statistically analyzed water quality parameters.

Village	F^−^	Ca^2+^	Mg^2+^	Na^2+^	K^+^	${\mathrm{NO}}_{3}^{-}$	${\mathrm{NO}}_{2}^{-}$	${\mathrm{SO}}_{4}^{2-}$	Cl^−^	EC	Fe	Mn
	(mg/L)	(mg/L)	(mg/L)	(mg/L)	(mg/L)	(mg/L)	(mg/L)	(mg/L)	(mg/L)	(μmhos/cm)	(mg/L)	(mg/L)
Ashtijeh 1	5.5	276	7.29	153.044	15	80	0.02	190	400	5370	0.02	0.01
Ashtije 2	5	132	43.74	221.158	10	32	0.02	190	475	4600	0.03	0.002
Khavas kuoh	1.6	66.8	39.609	169.008	12	52	0.01	185	230	1552	0.01	0.003
Malek kuoh	4	110	27.945	202.469	8.4	22	0.02	180	350	2300	0.01	0.003
Fakhr abad	5	156	63.18	167.319	12	80	0.02	175	425	4500	0.02	0.002
Sarsofla	4.8	132	102.1	145.276	15	40	0.02	190	500	4000	0.02	0.003
Chah shahi	4.4	120.8	31.104	257.853	11	27.2	0.01	195	475	2820	0.02	0.005
Zerjou	1.2	91.2	17.496	152.571	4.9	28.4	0.02	190	220	1540	0.03	0.003
Oghda(Qanat)	5.6	114.8	49.329	198.164	5.3	31.6	0.02	200	400	3150	0.03	0.002
Shams abad	5.1	200	87.48	499.014	10	36	0.01	175	475	4530	0.02	0.003
Sefid kuoh	5.6	304	46.17	106.226	13	39.2	0.05	430	375	4810	0.05	0.003
Haftadar	5	180	72.9	337.246	5	34.8	0.06	280	300	4080	0.09	0.003
zerjou(Qanat)	6	160	58.32	216.036	15	16	0.03	620	220	4700	0.05	0.005
Oghda 2	1	40	14.58	216.237	5	17.2	0.03	175	225	1500	0.02	0.001
Saghand	1.3	112	42.525	512.097	14	18	0.02	740	195	5000	0.06	0.007
Robat posht bam	4.6	49.2	19.926	150.588	3.1	20.4	0.03	148	155	1230	0.02	0.003
Maghestan	1.3	50	25.515	421.049	2.4	20	0.02	70	76	650	0.03	0.002
Diuok	1.1	76	38.88	218.974	5.8	28.8	0.03	195	250	2900	0.02	0.001
Robat	4.7	68	85.05	556.875	4.9	13.6	0.02	190	200	2600	0.06	0.004
Maghestan2	1.3	116	38.88	145.939	5.2	10	0.01	190	300	2680	0.02	0.003
Tuot	1.5	36	17.496	101.451	3.5	18.5	0.06	90	100	520	0.01	0.002
Kharanegh	0.9	72	75.33	963.195	3.1	17.1	0.02	195	150	4800	0.01	0.001
Tork abad 1	1	60	87.48	945.936	4	10.5	0.04	200	210	4000	0.03	0.007
Tork abad 2	0.9	52	89.91	142.56	3.6	12	0.01	185	300	2890	0.04	0.001
Tork abad 3	1.3	46	8.019	249.579	3	30	0.03	170	250	2500	0.03	0.001
Haji abad	1.2	29.6	5.346	512.962	3.6	12.5	0.01	70	45	500	0.05	0.001
Hamane	0.2	52	21.87	273.723	5.1	20.1	0.01	200	350	2410	0.02	0.002
Haji abad zarin	1	92	41.31	192.324	3.5	22	0.02	210	340	2450	0.01	0.003
Mean	2.9	106.9	44.96	164.87	7.37	28.2	0.02	222.4	285	3020.79	0.03	0.003
Max	6	304	102.1	512.1	15	80	0.06	740	500	5370	0.09	0.01
Min	0.2	29.6	5	34	2.4	10	0.01	70	45	500	0.01	0.001
S.D	2	68.87	28.33	96.62	4.35	17.8	0.01	144.8	126	1473.12	0.02	0

**Table 2:** Pearson's correlation coefficient.

	F^−^	Ca^2+^	Mg^2+^	Na^+^	K	${{\mathrm{NO}}_{3}}^{-}$	${{\mathrm{NO}}_{2}}^{-}$	${\mathrm{SO}}_{4}^{2-}$	Cl^−^	EC	Fe	Mn
F^−^	1											
Ca^2+^	0.711**	1										
Mg^2+^	0.266	0.181	1									
Na^+^	−0.157	−0.062	−0.27	1								
K^+^	0.585**	0.695**	0.173	0.351	1							
${{\mathrm{NO}}_{3}}^{-}$	0.478*	0.6**	−0.038	−0.017	0.577**	1						
${{\mathrm{NO}}_{2}}^{-}$	0.153	0.175	0.006	−0.221	-0.114	−0.024	1					
SO_4_	0.206	0.387*	0.168	0.59**	0.559	−0.066	0.152	1				
Cl^−^	0.554**	0.6**	0.316	0.172	0.548**	0.507**	−0.225	0.043	1			
EC	0.498**	0.724**	0.521**	0.205	0.628**	0.399*	0.055	0.565**	0.563	1		
Fe	0.252	0.225	0.239	−0.069	0.057	−0.158	0.341	0.481**	−0.162	0.239	1	
Mn	0.309	0.476*	0.037	0.222	0.536**	0.277	0.028	0.432*	0.163	0.468*	0.149	1

** Correlation is significant at the 0.01 level (2-tailed).

* Correlation is significant at the 0.05 level (2-tailed).

**Fig. 4:**
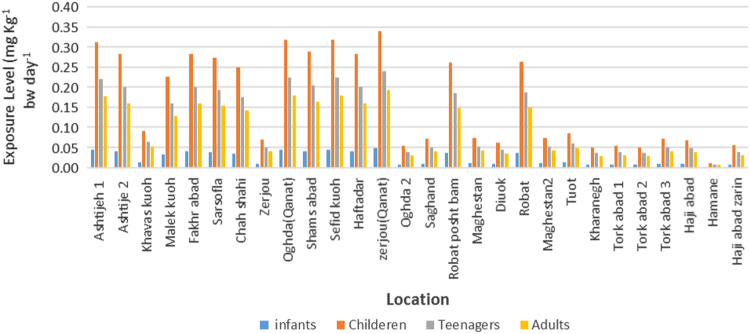
Fluoride exposure levels for different regions of Ardakan city over four age groups (infants, children, teenager and adults).

**Fig. 5:**
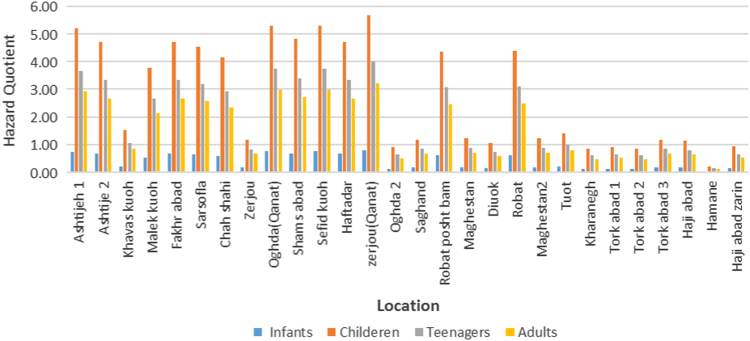
Hazard quotient value for different regions of Ardakan city over four age groups (infants, children, teenager and adults).

Fluoride exposure levels for different rural population was observed in four age groups as Fig. 4. Also, the HQ value for young groups was higher than 1 in Fig. 5.

## 2 Experimental design, materials and methods

### 2.1 Study area description

This study was conducted during September and December 2015 in Ardakan city of Yazd province (Fig. 1). Based on the Demographic Information of Iran, this city is populated with almost 77,758 with an area of 23,806 square kilometers that is located in 64 km North West of Yazd province. Ardakan city has a dry climate with an average temperature of 32.5 °C, an average annual rainfall of 58 mm and 2270 mm evaporation annually. Existence of mentioned industries plays a significant role in water contamination of this region via fluoride.

### 2.2 Determination of the water fluoride concentration

The samples were collected from drinking groundwater resources including wells and aqueducts from 28 villages of the city. A total of 112 samples were collected every four seasons over year from September- December 2015.

All water samples were analyzed according to the Standard Methods for Examination of Water and Wastewater. Also using titration method, temporary and permanent hardness, magnesium, calcium, and chloride were measured. Electrical conductivity was also analyzed with turbidity meter (model Hach 50161/co 150 model P2100Hach, USA). On the other hand, using Hach DR5000 spectrophotometer nitrate, and sulfate were determined compared with internal standards. Sampling was conducted with one‑liter polyethylene bottles which were immersed in nitric acid for 24 h then washed with 10 percent HCL and finally washed with distilled water. It has to be mentioned that before the collection of the samples, sampling containers had been rinsed at least three times with water. Fluoride concentration of collected samples was determined using SPADNS method according to Standard instruction [1], [2], [3], [4], [5], [6], [7], [8], [9], [10]. Then we assessed the fluoride concentration using Spectrophotometer (DR/5000, USA). Pearson's correlation coefficient was used for comparisons of association between all physicochemical parameters and data analysis was done using Excel 2016 software.

## 3 Risk assessment of fluoride

In order to understand the probability of adverse health effects it is beneficial to assess the health related risk of chemicals in contaminated water. Risk assessment is often the first step in safeguarding safety and health. In present study we used empirical models proposed by USEPA (1989) to estimate the non-carcinogenic effects of subjected contaminants [11], [12], [13], [14], [15], [16]. So, we quantitatively assessed the health related risk of fluoride through drinking water consumption in villages of Ardakan city, Yazd Province. Tap water samples were collected from different villages to meet the requirements of our study. In accord with same study (Mahmood Yousefi et al.) we divided population into four age groups based on physiological and behavioral differences including: infants (less than 2 years), children (2 to <6 years), teenagers (6 to <16 years) and adults (≥ 16 years). Also using following equation, the daily exposure dose of fluoride through water ingestion was measured [15]:

(1) $$\mathrm{EDI}=\frac{C_{f}\times C_{d}}{B_{w}}$$

Estimated Daily Intake (EDI) of fluoride is calculated based on the daily average consumption of drinking water (Cd), concentration of fluoride in drinking water (Cf) and body weight (Bw). EDI is expressed in unit of milligrams per kilogram of bodyweight per day. The data of water consumption and body weight were gathered via a questionnaire that was asked of the target groups (infants, children, teenager and adults). The average water consumption rates in infants (0–2 years old), children (2–6 years old), teenagers (6–16 years old) and adults (≥16 years old) were 0.08, 0.85, 2 and 2.5 L day^−1^, respectively. Body weight of target groups was considered 10, 15, 50 and 78 kg, respectively.

Hazard Quotient (HQ) means the ratio of a single substance exposure level (dose or concentration) over a specified period of time to the RfD or RfC derived for the same period of time for the same substance. A ratio larger than unity suggests that the concentration of the chemical is high enough to cause chronic noncarcinogenic effects.

Hazard quotient (HQ), an estimate of non-carcinogenic risks from exposure to fluoride through different exposure route, was calculated using following equation. Hazard quotient (HQ) is calculated by dividing the estimated daily intake (EDI) by the safe dose (RfD) [Eq. (2)]; in present study, we represent the fluoride intake risk from drinking water by HQ [16], [17], [18]:

(2) $$\mathrm{HQ}=\frac{\mathrm{EDI}}{\mathrm{RfD}}$$

The reference dose (RfD), is an estimate of a daily exposure to the human population over a lifetime without a considerable risk of deleterious effects. According to the Integrated Risk Information System, USEPA (USEPA, IRIS U), the oral reference doses of fluoride is 0.06 mg kg^−1^ d^−1^ [15], [16]. As it is mentioned previously, the HQ is the ratio between the EDI and RfD and HQ value less than one indicates that even for sensitive populations it is unlikely to experience adverse health effects. Whereas, when the value of HQ is exceeded 1, it well be understood that the adverse health effects are possible and the non-carcinogenic risk excesses the acceptable level.
